#  The Synthesis of GABA during the Tailbud Stage Is Required for Axial Elongation in *Xenopus laevis* embryos 

**DOI:** 10.17912/micropub.biology.001448

**Published:** 2024-12-30

**Authors:** Masaki Hachiman, Hiroki Kuroda

**Affiliations:** 1 Graduate School of Media and Governance, Keio University, Kanagawa, Japan; 2 Faculty of Environment and Information Studies, Keio University, Kanagawa, Japan

## Abstract

In *Xenopus laevis* , axial elongation beyond the tailbud stage requires gamma-aminobutyric acid (GABA). However, the role of GABA synthesized during early development in this process remains unclear. In this study, by treating embryos with allylglycine (AG), an inhibitor of GABA synthesis, we observed a significant reduction in axial elongation. This inhibition was rescued by exogenous GABA, demonstrating that GABA synthesis via glutamate decarboxylase (GAD) is essential for axial elongation after the tailbud stage. Our findings suggest that GABA-dependent elongation functions independently of mechanisms like convergent extension, which are crucial during early development.

**Figure 1. AG treatment inhibits axial elongation, and GABA rescues this inhibition:**
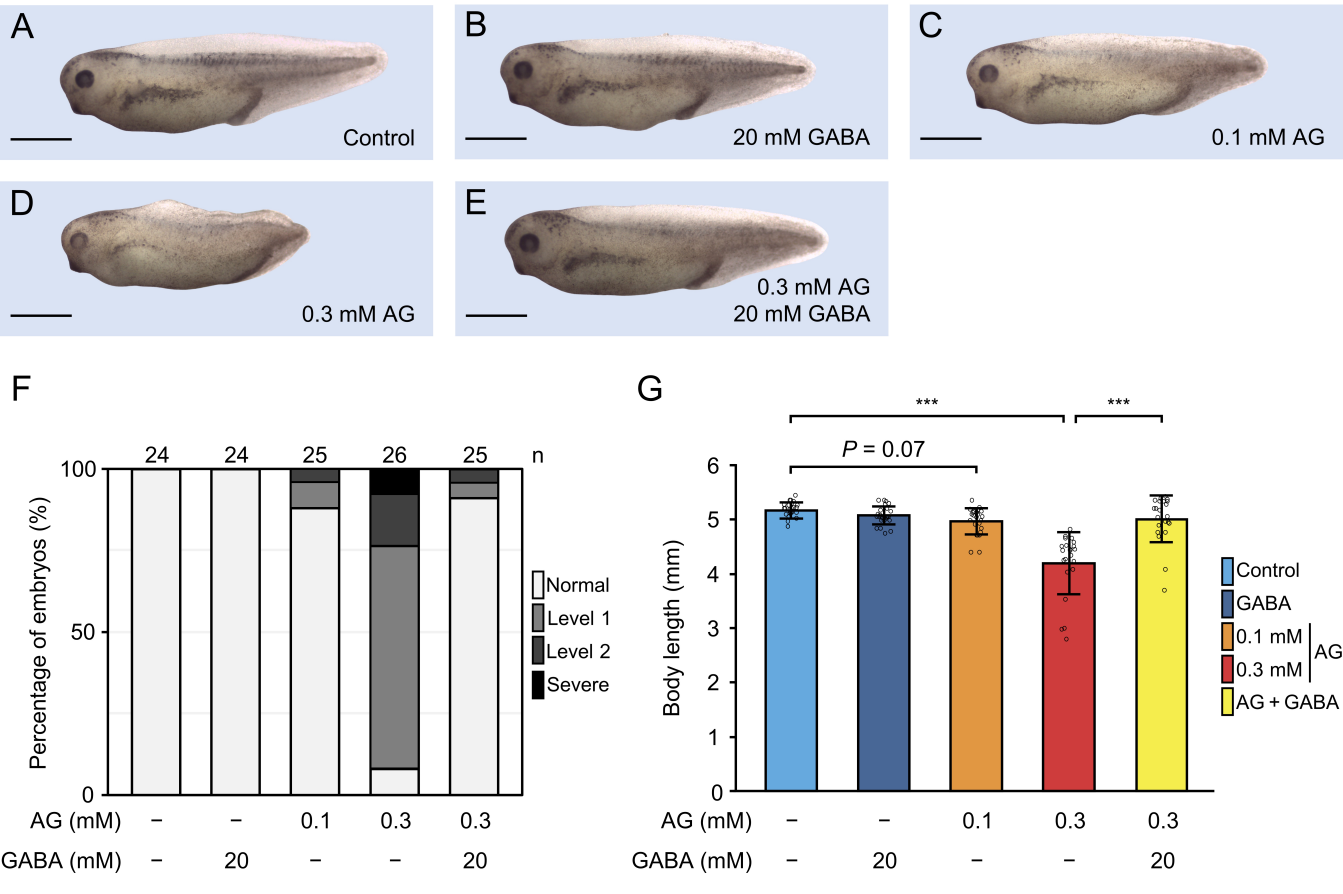
(A-E) Effects of AG and GABA treatments on embryos from St. 18 to 38. Scale bars: 1 mm. (A) Control embryo. (B) Embryo treated with 20 mM GABA. (C) Embryo treated with 0.1 mM AG. (D) Embryo treated with 0.3 mM AG. (E) Embryo treated with 0.3 mM AG and 20 mM GABA. (F) Quantification of AG-induced elongation inhibition. Embryos with body lengths ≥90% of the mean control length were classified as Normal, those with lengths between 75% and 89% as Level 1, and those <75% as Level 2. (G) Body lengths of St. 38 embryos. Data are presented as mean ± SD, with all measurements plotted. Statistical analyses were performed using one-way ANOVA followed by Tukey’s test. *** *P* ≤ 0.001 compared with control.

## Description

 In *Xenopus laevis* , a well-established vertebrate model organism, early embryos measure approximately 1.3 mm in length but elongate to about 2 mm during the early tailbud stage through convergent extension (Shindo, 2018) . Subsequently, the body axis continues to elongate without external nutritional input. However, the mechanisms driving elongation during the tailbud stage remain largely unexplored. 

 GABA, synthesized via the decarboxylation of L-glutamate by glutamic acid decarboxylase (GAD), is known as an inhibitory neurotransmitter in both vertebrate and invertebrate nervous systems (Owens and Kriegstein, 2002) . Transcriptomic studies have revealed that in amphibian embryos, GAD is expressed from early developmental stages—stage (St.) 10 in *X. laevis* embryos and St. 22 in the closely related *X. tropicalis* (Owens et al., 2016; Session et al., 2016) . *In situ* hybridization has shown overlapping expression of GAD and GABA receptor genes in neural regions, with metabolomic analyses detecting GABA nearby (Kaeser et al., 2011; Furukawa et al., 2019) . 

 Previous research demonstrated that treating *X. laevis* embryos with pentylenetetrazole (PTZ) and picrotoxin (PTX), competitive inhibitors of the GABA receptor GABA _A_ , inhibits axial elongation (Furukawa et al., 2019) . However, whether GABA synthesis during early development contributes to body axis elongation remains unclear. This study focuses on the role of GABA synthesis during early embryogenesis and its effects on elongation. Elevated GABAergic activity during the tailbud stage suggests a crucial role in tail elongation, whereas inhibition of the GABA _A_ receptor during other stages has no significant impact. The tail-forming region becomes fate-determined at approximately St. 18 (Tucker and Slack, 1995) . 

 To investigate GABA's role in embryonic development, St. 18–38 embryos were treated with allylglycine (AG), a GAD inhibitor, to block GABA synthesis. AG's structural similarity to L-glutamic acid makes it a commonly used GAD inhibitor and a known agent for inducing seizures (Horton et al., 1978; Taberner, 1977) . Using established evaluation criteria (Furukawa et al., 2019) , embryos were categorized as "Normal" if body length was ≥90% of controls, "Level 1" if 75–89%, and "Level 2" if <75%. 

 Results showed significant elongation inhibition in AG-treated embryos compared to controls, primarily affecting posterior development ( Figure 1C, 1D ). In embryos treated with 0.3 mM AG, 68% exhibited Level 1 elongation inhibition, 16% Level 2, and several failed to reach St. 38 ( Figure 1F ). Treatment with GABA alone did not significantly affect elongation ( Figure 1B, 1F ). However, co-treatment with AG and GABA rescued elongation inhibition ( Figure 1E, 1F, 1G ). Embryos treated with 0.3 mM AG had significantly shorter body lengths than controls ( *P* ≤ 0.001) ( Figure 1G ). 

 In conclusion, AG inhibits axial elongation by suppressing GABA synthesis via GAD, indicating that GABA synthesis during early development is essential for axial elongation post-tailbud stage. This process appears distinct from gastrulation-associated elongation mechanisms and is likely linked to secondary neurulation during the tailbud stage (Beck, 2015) . These findings suggest that GABA-induced axial elongation operates independently of established mechanisms such as convergent extension. 

## Methods

**AG and GABA treatments of embryos**

*X. laevis* embryos were obtained via in vitro fertilization, as detailed in a previous study (Ohata et al., 2014) . AG powder (58.95 mg; #A1648, Tokyo Chemical Industry Co., Japan) was dissolved in 300 μl of ultrapure water to prepare a 1 M stock solution. Similarly, GABA powder (103.12 mg; #G0048, LKT Labs, USA) was dissolved in 1 ml of 0.1× Steinberg’s solution (SS) to create a 1 M stock solution. Each stock solution was diluted with 0.1× SS to the desired concentration for experiments. To prevent embryos and explants from adhering to plastic plates, Poly-HEMA-coated plates were prepared. A 12% Poly-HEMA solution (#18894, Polysciences, USA) was diluted to 4% with ethanol. Then, 500 μl of the 4% solution was evenly spread on φ40-mm plastic plates, removed immediately, and allowed to dry. AG and GABA treatments were conducted in these Poly-HEMA-coated plates with 5 ml of the respective solution. Embryos were exposed to the solutions from St. 18 to 38. 

**Body length measurement**

Embryos were photographed under a stereomicroscope (#EZ4 HD, Leica, Germany). Body length was measured as the straight-line distance from the head apex to the tail tip using ImageJ software (version 1.52a, National Institutes of Health, USA).

**Statistical analysis**

 Data are presented as the mean ± standard deviation (SD), and all measurements were plotted. Statistical analyses were performed using one-way ANOVA followed by Tukey’s test. *P < 0.05* was considered significant. Statistical analyses were performed with EZR (Jichi Medical University, Japan; Kanda, 2013), which is a graphical user interface for R (The R Foundation for Statistical Computing, Austria). More precisely, it is a modified version of R commander (version 1.68) designed to add statistical functions frequently used in biostatistics. 

## References

[R1] Beck CW (2014). Development of the vertebrate tailbud.. Wiley Interdiscip Rev Dev Biol.

[R2] Furukawa T, Yamasaki Y, Hara Y, Otsuki C, Maki H, Soga T, Moriyama Y, Kuroda H (2019). Axis elongation during Xenopus tail-bud stage is regulated by GABA
expressed in the anterior-to-mid neural tube.. Int J Dev Biol.

[R3] Horton RW, Chapman AG, Meldrum BS (1978). Regional changes in cerebral GABA concentration and convulsions produced by
D and by L-allylglycine.. J Neurochem.

[R4] Kaeser GE, Rabe BA, Saha MS (2011). Cloning and characterization of GABAA α subunits and GABAB subunits in
Xenopus laevis during development.. Dev Dyn.

[R5] Kanda Y (2012). Investigation of the freely available easy-to-use software 'EZR' for
medical statistics.. Bone Marrow Transplant.

[R6] Ohata Y, Matsukawa S, Moriyama Y, Michiue T, Morimoto K, Sato Y, Kuroda H (2014). Sirtuin inhibitor Ex-527 causes neural tube defects, ventral edema
formations, and gastrointestinal malformations in Xenopus laevis
embryos.. Dev Growth Differ.

[R7] Owens DF, Kriegstein AR (2002). Is there more to GABA than synaptic inhibition?. Nat Rev Neurosci.

[R8] Owens NDL, Blitz IL, Lane MA, Patrushev I, Overton JD, Gilchrist MJ, Cho KWY, Khokha MK (2016). Measuring Absolute RNA Copy Numbers at High Temporal Resolution Reveals
Transcriptome Kinetics in Development.. Cell Rep.

[R9] Session AM, Uno Y, Kwon T, Chapman JA, Toyoda A, Takahashi S, Fukui A, Hikosaka A, Suzuki A, Kondo M, van Heeringen SJ, Quigley I, Heinz S, Ogino H, Ochi H, Hellsten U, Lyons JB, Simakov O, Putnam N, Stites J, Kuroki Y, Tanaka T, Michiue T, Watanabe M, Bogdanovic O, Lister R, Georgiou G, Paranjpe SS, van Kruijsbergen I, Shu S, Carlson J, Kinoshita T, Ohta Y, Mawaribuchi S, Jenkins J, Grimwood J, Schmutz J, Mitros T, Mozaffari SV, Suzuki Y, Haramoto Y, Yamamoto TS, Takagi C, Heald R, Miller K, Haudenschild C, Kitzman J, Nakayama T, Izutsu Y, Robert J, Fortriede J, Burns K, Lotay V, Karimi K, Yasuoka Y, Dichmann DS, Flajnik MF, Houston DW, Shendure J, DuPasquier L, Vize PD, Zorn AM, Ito M, Marcotte EM, Wallingford JB, Ito Y, Asashima M, Ueno N, Matsuda Y, Veenstra GJ, Fujiyama A, Harland RM, Taira M, Rokhsar DS (2016). Genome evolution in the allotetraploid frog Xenopus laevis.. Nature.

[R10] Shindo A (2017). Models of convergent extension during morphogenesis.. Wiley Interdiscip Rev Dev Biol.

[R11] Taberner PV, Keen P (1977). Brain and blood levels of allylglycine in mice following doses sufficient
to inhibit glutamate decarboxylase.. J Neurochem.

[R12] Tucker AS, Slack JM (1995). Tail bud determination in the vertebrate embryo.. Curr Biol.

